# Evaluating *LRRK2* Genetic Variants with Unclear Pathogenicity

**DOI:** 10.1155/2015/678701

**Published:** 2015-03-02

**Authors:** Fathima Shaffra Refai, Shin Hui Ng, Eng-King Tan

**Affiliations:** Department of Neurology, National Neuroscience Institute, 7 Hospital Drive, Outram Road, Singapore 169611

## Abstract

Mutations in the leucine-rich repeat kinase 2 (LRRK2) have been known to be a major genetic component affecting Parkinson's disease (PD). However, the pathogenicity of many of the *LRRK2* variants is unclear because they have been detected in single patients or also in patients and controls. Here, we selected 5 exonic variants (L1165P, T1410M, M1646T, L2063X, and Y2189C) from each of the protein domain of *LRRK2* and analysed their possible association with pathogenicity using *in vitro* functional assays. Point mutations representing each of these variants were incorporated into the *LRRK2* gene, and functional aspects such as the percentage of cell survival upon application of stress and kinase activity were measured. Our results showed that all 5 variants had a significantly negative effect on the survival of cells, in both presence and absence of stress, as compared to the wild-type. In addition, there was also a slight increase in kinase activity in most of the variants in comparison to the wild-type. A negative correlation between cell survival and kinase activity was observed. These data suggest that most of the variants despite being located in different domains of *LRRK2* appear to exert a potential pathogenic effect possibly through an increased kinase activity, supporting a gain of function mechanism.

## 1. Introduction

Over the past decade, several genes have been linked to the pathogenesis of Parkinson's disease (PD), a neurodegenerative disorder characterized by the loss of dopaminergic neurons in the substantia nigra pars compacta of the brain [1]. Among these genes, mutations in leucine-rich repeat kinase 2 (*LRRK2; PARK8*; MIM number 609007) are the most common cause of Mendelian inheritance of PD, contributing to at least 5% of familial PD patients [2]. Interestingly, mutations in* LRRK2 *have also been found in 1-2% of sporadic PD cases, narrowing the lines distinguishing between sporadic and genetic causes of the disease [3]. Polymorphic LRRK2 variants have also been shown to modulate risk of PD [4–7].

The* LRRK2* gene is encoded by a segment containing 51 exons, located in the chromosome 12q12 locus, which is highly conserved among most vertebrates [8]. It encodes a large 2527-amino-acid protein belonging to the ROCO family [8, 9] and is composed of several distinct domains: leucine-rich repeat (LRR), Roc (Ras in complex proteins) [10], COR (C-terminal of ROC), tyrosine and serine/threonine kinase (MAPKKK), and WD40 domain. Even though the precise role of LRRK2 is yet to be understood, the presence of these domains indicates its participation in cell signaling pathways [11].

To date, up to 7649 different* LRRK2* sequence variants have been identified and reported worldwide, from many different ethnicities (http://www.ncbi.nlm.nih.gov/projects/SNP/). However, only 6 of these variants (N1437H, R1441C, R1441G, Y1699C, G2019S, and I2020T) are considered to be definitely disease causing, based on their absence in controls and cosegregation of the disease in families [12]. The role of many other variants and their contribution to the disease is currently unknown. One of the key questions is whether these variants located in different protein domains exert their pathogenicity (if any) via an increased kinase activity. This has important implications in maximizing therapeutic approaches in* LRRK2*-linked PD.

To address these gaps in knowledge, we selected 5* LRRK2* variants, one from each domain, whose pathogenicity is unclear because they are present in single patients or in patients and controls. These could be rare polymorphic variants, benign or genetic mutations. We designed an* in vitro* cell-based system, expressing the 5 variants, and analysed their toxicity using kinase and cell survival assays.

## 2. Materials and Methods

### 2.1. *LRRK2* Expression Constructs

**Figure 1:**
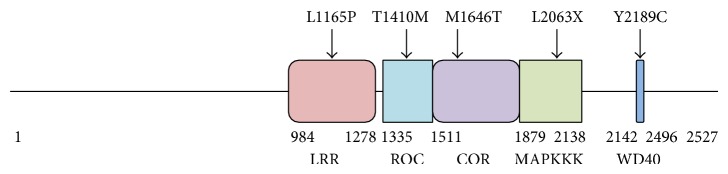
Schematic representation of LRRK2 protein, showing variants reported in patients with PD in relation to the domains.

The* pEGFP-N1-LRRK2-WT* plasmid coding for the human* LRRK2* cDNA (GenBank: BC117180.1) sequence was used as a template to generate point mutations, 3494T>C (L1165P), 4229C>T (T1410M), 4937T>C (M1646T), 6187_6191delCTCTA (L2063X), and 6566A>G (Y2189C) (Figure 1), using the QuickChange site-directed mutagenesis kit (Stratagene) in accordance with the manufacturer's protocol. The authenticity of the resulting plasmids was confirmed via sequencing using a sequencer (ABI PRISM 3100 Genetic Analyzer; Applied Biosystems).

### 2.2. Cell Culture and Transfection

Human embryonic kidney 293T (HEK 293T) cells were cultured in Dulbecco's modified Eagle's medium (DMEM; Invitrogen) supplemented with 10% fetal bovine serum, 100 U/mL penicillin, and 100 mg/mL streptomycin, 1X nonessential amino acids, and 100 mg/mL sodium pyruvate (all from Gibco-BRL) at 37°C in a 5% CO_2_ incubator. The plasmids were transfected using Lipofectamine 2000 (Invitrogen) with reference to the manufacturer's protocol.

### 2.3. Treatment with H_2_O_2_

HEK 293T cells transiently expressing wild-type and mutant forms of LRRK2 were incubated in culture medium containing 500 *μ*M H_2_O_2_ for 24 hours. The optimal concentration for the treatment of HEK 293T cells was determined by investigating the H_2_O_2_ concentration that would elicit a cell death of 50% (EC_50_) from a dose-response curve.

### 2.4. Analysis of Cell Survival

Upon treatment with H_2_O_2_, cell viability was assessed using Cell Titer 96 AQ_ueous_ One Solution Reagent (MTS Assay; Promega). Cells were incubated with MTS/DMEM solution (1 : 5 ratio) at 37°C for 1.5 hours in the dark. The resulting solution was mixed well and the optical density (OD) measured at 490 nm using a spectrophotometer (Benchmark plus, Microplate spectrophotometer; Biorad). Results were expressed as a percentage of untreated wild-type control.

### 2.5. Assessment of Kinase Activity

*In vitro* LRRK2 kinase activity was measured using Transcreener ADP^2^ FI Assay (BellBrook Labs). LRRK2 variants were purified using agarose anti-GFP beads (Vector Laboratories). The purified proteins were incubated in 25 *μ*L of kinase buffer (15 mM HEPES, 20 mM NaCl, 1 mM EGTA, 0.02% Tween 20, 10 mM MgCl_2_, and 0.1 mg/mL BGG) containing 100 *μ*M ATP for 1 hour at 30°C in a black opaque 96-well plate. The reactants were then mixed in a ratio of 1 : 1 with ADP Detection Mixture containing 1X Stop & Detect buffer B, 8 nM ADP Alexa594 Tracer, and 94 *μ*g/mL ADP^2^ Antibody-IRDye QC-1, and incubated at room temperature for 30 minutes. The intensity of fluorescence emission was measured using the Synergy H1 Hybrid Microplate reader (Biotek, USA) at a wavelength of excitation of 590 nm and emission of 617 nm.

### 2.6. Statistical Analysis

The statistical significance was assessed using the two-tailed Student's* t*-test, with a 1% level of significance to test the hypothesis. Correlation coefficients between kinase and cell survival data were used to analyse the significance of their relatedness.

## 3. Results

HEK 293T cells transiently expressing wild-type or mutant forms of LRRK2 were created. These include those expressing L1165P, T1410M, M1646T, L2063X, Y2189C, or wild-type LRRK2 (Figure 1).

### 3.1. Baseline Cell Survival

**Figure 2:**
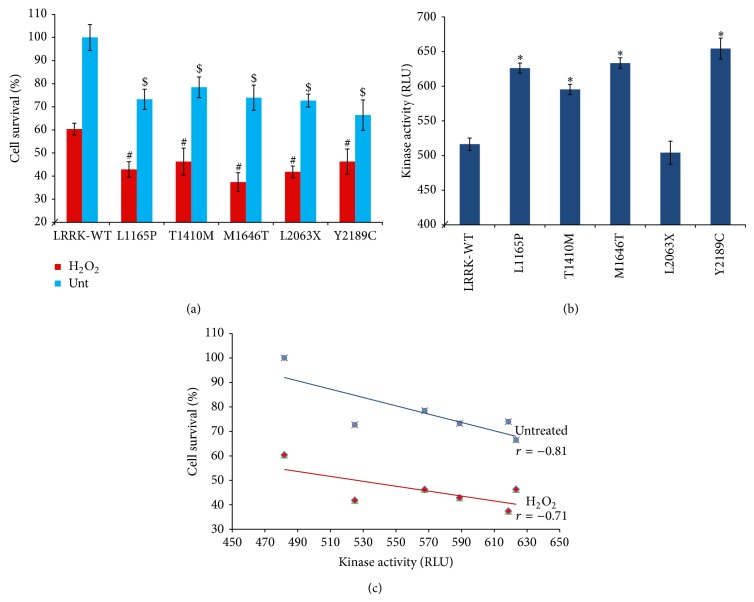
Impact of LRRK2 wild-type and mutants on basal cell survival and survival in response to H_2_O_2_ insult in HEK 293T cells and their corresponding kinase activities. (a) Changes in percentage cell survival between cells expressing LRRK2 wild-type and mutants from LRR (L1165P), ROC (T1410M), COR (M1646T), kinase (L2063X), and WD40 (Y2189C) domains, shown in response to 500 *μ*M H_2_O_2_ for 24 hours. (b) Autokinase activity of the wild-type and mutants in RLU measured using the Transcreener ADP^2^ FI Assay. (c) Correlation between autokinase activity and percentage cell survival. Data are means ± standard error of the mean (SEM), three readings per data point, from at least 3 independent experiments. Two-tailed Student's* t*-test; ^#^ *P* < 0.005 and ^$^ *P* < 0.005 versus percentage change in cell survival in cell expressing LRRK2 mutants with respect to the wild-type, in the presence and absence of H_2_O_2_, respectively; ^*^ *P* < 0.005 versus kinase activity with respect to wild-type LRRK2.

Cell lines expressing all mutants exhibited a significantly lower percentage of cell survival as compared to the wild-type LRRK2, in the absence of any form of stress (*P* < 0.001) (Figure 2(a)). Mutant Y2189C from the WD40 domain induced the most amount of toxicity exhibiting a mean cell survival of only 66%. On average, cell survivability of all mutants ranged between 66 and 78%.

### 3.2. Cell Survival upon H_2_O_2_ Application

Similarly, upon application of stress induced by H_2_O_2_, cell lines expressing the mutant forms of LRRK2 exhibited a significantly lower amount of cell survival than that of the wild-type (*P* < 0.001) (Figure 2(a)). The highest toxicity was induced by the COR domain mutant M1646T (cell survival = 37%), while the least toxicity was induced by the WD40 domain mutant Y2189C (cell survival = 46%).

### 3.3. Kinase Activity

Experiments depicting the autokinase activity of LRRK2 variants showed a significantly increased activity in all variants as compared to wild-type (*P* < 0.001), except for L2063X (*P* = 0.007) (Figure 2(b)). L2063X is a truncation mutation in the kinase domain, and these results suggest that amino acids 1879 to 2063 are enough to maintain baseline kinase activity.

### 3.4. Correlation between Kinase Activity and Cell Survival

Correlation coefficient calculations showed a strong negative correlation between autokinase activity and percentage of cell survival in the absence (*r* = −0.81) and presence of stress (*r* = −0.71) (Figure 2(c)).

## 4. Discussion

Extensive research has been carried out to identify the numerous* LRRK2 *variants in PD. Although commonly occurring mutations, for example, the G2019S, highly frequent (30–40%) among the Ashkenazi Jews and the North African Arabs [20], have been well-studied, there is limited information available on most of the other variants [1, 21]. One of the key questions is whether these variants located in different protein domains exert their pathogenicity (if any) via an increased kinase activity. Development of specific LRRK2 kinase inhibitors has been the current key approach worldwide.

Recently, Ross et al. [15] in a case-control study assessed 121 exonic variants in 15,540 individuals (8611 patients and 6929 controls) from the white, Asian, and Arab-Berber populations. They identified risk associations of variant M1646T in white, A419V and G2385R in Asian, and Y2189C in Arab-Berber populations. In our study, we tested for the risk of carrying two of these variants (M1646T and Y2189C) together with some others (L1165P, M1646T, and L2063X) using functional assays. The selection criteria were based on the fact that these variants have been detected in single patients or also in patients and controls, and there have been no functional studies previously reported on these variants; hence, their pathogenicity is unclear. We selected one variant from each representative protein domain (Figure 1).

Our results from the cell survival and kinase studies suggest that all of these variants appear to be toxic, and carrying any of them would likely increase the risk of developing PD (Figure 2). The strong negative correlation between kinase activity and cell survival is consistent with previous findings showing that increased autokinase activity of LRRK2 G2019S is harmful to the cell [22, 23]. Smith et al. [22] reported a 3-fold increase in LRRK2 G2019S autokinase activity in comparison to wild-type, contributing to a 2-fold decrease in cell viability, while, in our hands, we previously reported a 2-fold increase in LRRK2 G2019S autokinase activity as compared to wild-type, which was accompanied by a slight increase in neuronal cell toxicity [24]. In another instance, Chan et al. [25] also noted that neuronal cells transiently expressing LRRK2 G2019S are 1.1 times more toxic as compared with wild-type. It is also possible that LRRK2 mutants induce both kinase-dependent and independent forms of cell death and this should be addressed in further studies.

Nonkinase domains may have a regulatory effect on the kinase domain, either directly or indirectly via the interaction with other proteins. The ROC domain comprising of conserved motifs for GTPase activity is known to regulate kinase activity by acting as a molecular switch, alternating between GDP- and GTP-bound states [22, 26]. When bound to GTP, the switch region located outside the domain is in an active state, leading to an increase in kinase activity, while when bound to GDP, the tertiary structure of the switch region is in an inactive state, thereby leading to a decrease in kinase activity. These conformational changes in the ROC domain are conveyed to the kinase domain via the COR domain which acts as molecular hinge. LRR and WD40 domains are composed of highly conserved folds often found in signalling proteins in which they play a role in protein-protein interaction [27]. Variants in these domains may influence the LRRK2 kinase activity by mediating the interaction with its substrate, for example, by causing hyperphosphorylation of a neuroprotective protein. Jorgensen et al. [28] reported the involvement of WD40 domain in LRRK2 dimerization, known to affect its kinase activity and by that cell death.

Only one case of PD, with the L1165P mutation has been reported, where the patient developed the first signs of disease at the age of 47 (Table 1) [14]. The patient displayed symptoms typical of PD, together with dementia, and, pathologically, it was classified as a Lewy body disease [13, 14]. The absence of this substitution in all of the controls screened confirmed its pathogenicity [14]. L1165 is known to be highly conserved across many species, and therefore a substitution is predicted to cause a dramatic change structurally in the Leucine-rich region [14]. SIFT analyses predicted that the L1165P substitution is not tolerated.

There is ambiguity in the trend reported for the T1410M variant. Some have classified it to be nonpathogenic due to the fact that it is present in both cases as well as in controls (Table 1) [16], while others say that the mutation affects a highly conserved region, located in the ROC domain, and therefore may distort the tertiary structure of the protein, hence disrupting its GTP hydrolysis function [17, 26]. T1410 has also been identified as an autophosphorylation site by mass spectrometry [29, 30]. Our results together with SIFT analyses depict that T1410M tends more towards the pathogenic direction.

Not much has been mentioned about the variants M1646T and L2063X in published literature. L2063X is a truncation mutation in the kinase domain, but the kinase activity is not affected. The exact reason as to why it causes apoptosis is unclear. The Y2189C variant was first identified by Nuytemans et al. [19] in the Belgian population and he reported that* in silico* conservation analysis showed that the residue Tyr in itself was not evolutionarily conserved, but the aromatic nature of the amino acid was, and may therefore be important in maintaining the structure and function of the protein. They provided evidence from SIFT analyses that Y2189C is deleterious for the function of LRRK2. Other groups also reported its trend toward association due to an increase in the minor allele frequency in patients as compared to controls [18]. Our observation of the highest cellular toxicity being exerted by this mutant in the absence of H_2_O_2_, while the lowest toxicity in the presence of H_2_O_2_ depicts that WD40 domain may be less susceptible to oxidative stress.

Our study has limitations. First, we have only examined 5 variants and can only provide proof of principle findings specific to these variants only. Second, we have used autophosphorylation of LRRK2 as a surrogate marker of kinase activity. LRRK2 is likely to have physiologic substrates or protein interactors* in vivo* that can modulate its activity.

In conclusion, as proof of principle experiments, we investigated 5* LRRK2* variants (L1165P, T1410M, M1646T, L2063X, and Y2189C) with unclear pathogenicity located in each of the major protein domain. Our results suggest that these variants are proapoptotic and this appeared to correlate with an increased kinase activity in most variants. Further studies in animal models will be useful to further characterise their pathogenicity and this will potentially help future genetic testing programmes. Our study also suggests that some of the variants, even if they are not in the kinase domain, may act via a common toxic gain of function likely through an increased kinase activity. Specific LRRK2 inhibitors could have potential therapeutic uses in carriers of these variants.

## Figures and Tables

**Table 1:** *LRRK2* ^1^ mutations and associated clinical features.

Protein domain	Nucleotide change	Amino acid change	Number of patients	Number of controls	Reported clinical features	Source of information
Leucine-rich repeat (LRR)	3494T>C	L1165P	1	0	Age of onset around 47 years. Slow progressive disease with typical PD symptoms that respond well to levodopa therapy	Chen-Plotkin et al., 2008 [13]; Covy et al., 2009 [14]

Ras of complex proteins (ROC)	4229C>T	T1410M	19	11	Middle-age to late onset of disease, displaying typical symptoms of PD and a good response to levodopa therapy	Ross et al., 2011 [15]; Lesage et al., 2009 [16]; Abdalla-Carvalho et al., 2010 [17]

C-terminal of ROC (COR)	4937T>C	M1646T	4	2	Typical PD	Jasinska-Myga et al., 2010 [18]; Ross et al., 2011 [15]

MAP kinase kinase kinase (MAPKKK)	6187_6191delCTCTA	L2063X	1	2	Typical PD	Ross et al., 2011 [15]

WD40 repeat (WD40)	6566A>G	Y2189C	14	8	Middle-age to late onset of disease, showing characteristic PD symptoms with a positive response to levodopa therapy	Abdalla-Carvalho et al., 2010 [17]; Nuytemans et al., 2008 [19]; Jasinska-Myga et al., 2010 [18]; Ross et al., 2011 [15]

^1^GenBank: BC117180.1.
